# Clinical Evaluation of Short Tuberosity Implants among Type 2 Diabetic and Non-Diabetic Patients: A 5 Year Follow-Up

**DOI:** 10.3390/medicina58101487

**Published:** 2022-10-19

**Authors:** Huda I. Tulbah, Abdulaziz Alsahhaf, Hamad S. AlRumaih, Fahim Vohra, Tariq Abduljabbar

**Affiliations:** 1 Prosthetic Dental Science Department, College of Dentistry, King Saud University, Riyadh 11451, Saudi Arabia; 2 Department of Substitutive Dental Sciences, College of Dentistry, Imam Abdulrahman Bin Faisal University, Dammam 31441, Saudi Arabia; 3 Research Chair for Biological Research in Oral Health, College of Dentistry, King Saud University, Riyadh 11451, Saudi Arabia

**Keywords:** diabetics, short tuberosity implant, periodontal parameters, crestal bone loss

## Abstract

*Aim:* To assess clinical and radiographic parameters including bleeding on probing (BoP); probing depth (PD), plaque index (PI) and crestal bone loss (CBL) around short tuberosity implants (STI) supporting fixed partial dentures in patients with Type 2 diabetes mellitus (T2DM) and non-diabetics. *Material and Methods:* Participants with T2DM and without T2DM with at least one STI (6 mm) posteriorly restored with a fixed partial denture splinting premolar implant were included. A questionnaire collected demographic details including gender, age, duration of diabetes, habits of brushing, the total number of dental implants and location, implant loading after placement, restoration type, and family history of DM. Clinical and radiographic assessment of peri-implant parameters, i.e., bleeding on probing (BoP), probing depth (PD), plaque index (PI), and crestal bone loss (CBL) was performed. The restorative success of STI was determined by no sensation of the foreign body, lack of pain and dysesthesia, lack of infection, no radiolucency around the implant, and no mobility. The Kruskal–Wallis test was used for statistical analysis. A *p*-value of less than 0.05 was considered statistically significant. *Results:* Twenty-five T2DM (19 males and 6 females) and 25 non-diabetic (18 males and 7 females) participants were included. The number of STIs in T2DM was 41, whereas in non-diabetic it was 38. At 1 year follow-up, mean PI% in T2DM participants was 18.9% (19.2–21.4%) and in non-diabetics it was 17.6% (16.3–18.5%). The mean PD was recorded in diabetics (1.3 ± 5.0 mm) and non-diabetics (1.1 ± 3.2 mm). The BoP value in diabetics was 44.9% (39.8–46.4%) and 28.2% in non-diabetics (17.2–24.6%). At 5 years of follow-up, the mean PI% range in T2DM participants was 26.18% (25.4–29.1%) and 24.42% in non-diabetic (20.1–25.5%). The mean PD in millimeters around STI in T2DM was observed to be 2.3 ± 4.8 mm and 1.4 ± 3.4 mm in non-diabetics. In addition, BoP in diabetic participants was 39.54% (27.7–42.1%) and 24.42% in non-diabetics (20.1–25.5%). A total of six STIs failed, i.e., two in the non-diabetic and four in the T2DM group. *Conclusions:* Patients with T2DM have poor periodontal (BoP, PD, CBL) and restorative peri-implant parameters around STIs when compared to healthy (non-diabetic) participants at five years of follow-up. For long-term stability, glycemic control is pivotal along with following good plaque control.

## 1. Introduction

Dental implants are considered to be safe options for the loss of partial or complete dentition [1]. Implant survival is directly associated with stability and osseointegration [2]. These two factors are constructed on the mechanical aspect and biological response of the tissue [2]. This includes the quality and thickness of alveolar bone, the implant’s surface, anatomical variation, environment of the location where the implant is placed, prosthesis design, occlusion, and loading of the implant [3]. However, failure of implants occurs when they are positioned in compromised bone quality i.e., in an area of the maxillary molar or in patients with bone resorption [4]. Evidence advocates the survival rate of implants is between 90% to 100% [3,4]. However, the stability and osseointegration of implants are compromised in patients with diabetes mellitus (DM) [1,5].

DM is considered to be an endocrine disorder in which there is partial or complete unavailability of insulin [5]. Type 2 DM (T2DM) is the most common subtype. Uncontrolled T2DM compromises the immune response, reduces bone remodeling, delays healing, and diminishes general health [6,7]. Additionally, it is considered a contradiction for patients with implant treatment [8]. In individuals with T2DM, the incidence of peri-implant diseases i.e., peri-implant mucositis and peri-implantitis, is found to be considerably higher. Furthermore, these individuals show high plaque scores (PS), crestal bone loss (CBL), and probing depth (PD) [9,10].

Increased age along with chronic disease (T2DM) compromises the quality and quantity of alveolar bone [11]. The quantity of bone decides the length and width of implants [12], while osseointegration is associated with the quality of the bone. T2DM with age concessions both the quality and quantity of bone [13]. Since T2DM is a disease affecting the elderly, poor alveolar bone conditions with extensive healing times and delayed immune response is expected [14]. Microradiographic evidence demonstrates that in T2DM, with increasing age the cortical porosity increases considerably, which directly decreases bone mass that is noticeable more in women than men [15]. Recent work by Moy et al. showed the risk of implant failure increases with progressing age and patients with T2DM have a poor prognosis [16]. Along with T2DM, old patients have advanced bone loss especially over the sinuses. In cases where extensive surgery cannot be conducted to replace bone, an implant in the tuberosity area can enable a replacement of lost molars using implant-supported splinted restoration from the premolar to the tuberosity area [17].

To overcome this problem, the use of short tuberosity implants (STI) is a contemporary approach that minimizes damage to the vital structures and is easy to facilitate in areas of alveolar bone with low quantity and quality [18]. STI has the added advantage of low risk of surgical paresthesia, easy removal in case of failure, and less discomfort to patient cost and time [19]. These benefits seem to be a benediction in old age and T2DM patients. To date, there is no study that has gauged the clinical, radiographic, and periodontal parameters among T2DM and non-diabetic patients. It is hypothesized that STI will be a viable option in both T2DM and nondiabetic patients. Hence, the present study aimed to assess clinical and radiographic parameters including bleeding on probing (BoP), probing depth (PD), plaque index (PI) and crestal bone loss (CBL) around short tuberosity implants (STI) supporting fixed partial dentures in patients with Type 2 diabetes mellitus (T2DM) and non-diabetics.

## 2. Materials and Methods

### 2.1. Ethical Contemplations

The study protocol was reviewed and approved by the ethical committee of the center for specialist dental practice and clinical research (UDCRC-RB-033-21-) Saudi Arabia. The study followed the ethical guidelines of the Declaration of Helsinki (2013). All participants were made to sign a form containing the research objectives and techniques. All participants were allowed to exit the study at any point in time without any penalty or consequences.

### 2.2. Research Questionnaire and Criteria for Eligibility

The questionnaire was adopted from already-reported work by Alhenaki et al. [20]. The questionnaire consisted of 13 questions including demographic details, i.e., gender, age, duration of diabetes, habits of brushing, the total number of dental implants and location, implant loading after placement, restoration type, family history of DM, and oral and general health visits. The participants were included based on the following inclusion criteria: individuals with T2DM (HbA1c > 6.0%) and systemically healthy individuals without T2DM (HbA1c < 5.7%), patients with at least one STI posteriorly on either side of the maxilla (6 mm), restored with a multiple-unit splinted prosthesis with a follow-up of 5 years or more, the presence of a baseline post-operative radiograph, those with regular attendance to the dentist for supportive periodontal treatment and patients who were comfortable in signing the consent form. The participants were excluded based on the following exclusion criteria: subjects consuming alcohol or who had smoked tobacco, individuals having a systemic disease (hepatic, renal, cardiovascular), Human immunodeficiency syndrome (HIV) patients, bone grafting performed for implant placement, individuals on steroids and non-steroidal anti-inflammatory drugs (NSAIDs), those given antibiotics in the last 3 to 6 months and participants whose baseline and follow-up radiographs were not available [21,22].

### 2.3. Dental Implant Surgical and Prosthetic Protocol

An STI was placed by a trained oral and maxilla-facial surgeon based on planning from a prosthodontist. Each STI implant length was 6 mm and the antibiotic amoxicillin at a dose of 1 g was administered prophylactically the night before the surgical procedure. This was followed by the administration of 1.5 g amoxicillin post-operatively for seven days. Subjects who were allergic to penicillin were administered clindamycin 2 g pre and post-operatively. All subjects were given ibuprofen as an analgesic for six to eight hours. A daily rinse of chlorhexidine digluconate 0.2% was prescribed for all participants for two weeks twice daily starting from the day of the procedure. Using a number 15 surgical blade, the mucoperiosteal flap was raised under local anesthesia. A standard drilling sequence was used for dental implant osteotomy sites. To minimize the risk to the maxillary sinus, rubber stops were introduced on the drills, which were maintained 1 mm less than the radiographic working length. STIs (diameter of 4 mm and length of 8 mm) (ITI Straumann, Bern, Switzerland) at the level of soft tissue were positioned in all subjects at the regions of maxillary tuberosity on either side. Loading of the implant was performed after 10–12 weeks of being submerged. Screw-retained and cemented-retained porcelain fused to metal-fixed dental prostheses were placed after 12 to 16 weeks post-healing. The prostheses were connected from premolar to STI with pontics at the first and second molars. The prosthesis was supported by regular dimension implants at the premolar and an STI at the tuberosity behind the maxillary sinus. All implants placed in the tuberosity were used as part of a full mouth prosthesis connected to implants placed in the maxilla. Before implant treatment, all participants were provided supportive periodontal therapy (debridement) using an ultrasonic scaler with carbide tips (NewtronP5XS, Ultrasonic Scaler Unit, Acteon, Lombart, IL, USA). All patients were educated on maintaining proper oral hygiene when visited at each appointment. The sutures were removed one week after the surgical procedure [23,24,25].

### 2.4. Clinical and Radiographic Assessment of Peri-Implant

Bleeding on probing (BoP), probing depth (PD), plaque index (PI) and crestal bone loss (CBL) medially and distally were measured by an experienced periodontist and prosthodontist [26] who were blinded to the study groups. The overall mean κ scores for inter- and intra-examiner reliability were 0.85 and 0.88, respectively. Measurements were taken at baseline, 12 months, and 60 months as recommended and guided by the Consensus report of the Eleventh European Workshop on Periodontology [27]. Measurements (PI, PD, and BoP) were taken from six sites of an implant (mesiolingual, distolingual, and mid-lingual, and mesiobuccal, distobuccal, and mid-buccal). Peri-implant scoring for plaque index PI and BoP were based upon dichotomous recording as present= 1 and absent = 0 (presented as a percentage). PD was measured to the nearest millimeter (mm) whole number, from the gingival margin to the most apical gingival tissue penetration of the periodontal probe tip (UNC-15, Hu-Friedy, Chicago, IL, USA) [8]. Standardization of digital radiography was done using the long cone technique to measure CBL at 12 months and 60 months. No loss to follow-up was noted in the present study during the last 5 years. The linear distance was ascribed from the rough implant surface (junction of implant abutment) to the lower alveolar crest in mm. CBL is described as the median loss of bone at the peri-implant aspect distally and medially.

### 2.5. Restorative Parameters Assessment

The rate of success of the STI was determined using the criteria followed by Buser et al. [27]: (1) no sensation of the foreign body, and lack of pain or dysesthesia; (2) no infection around the dental implant; (3) no radiolucency around the implant surface; (4) no mobility. Other implant-related restorative parameters, i.e., loosening of the implant, fracture of the framework, crown chipping, abutment screw loosening, and fracture of the implant, were also recorded and evaluated

### 2.6. Statistical Analysis

The Software Program for Social Sciences (SPSS Version 24, Chicago, IL, USA) was used for statistical analysis. Numerical ranges and means are measured. The sample size and post hoc power for each group were assessed using nQuery Advisor 6.0 (Statistical Solutions, Saugus, MA, USA). Means and SDs among comparable groups were analyzed using the Kruskal–Wallis test (statistical analysis). A *p*-value less than 0.05 was considered statistically significant.

## 3. Results

### 3.1. General Characteristics of the Cohort

The total number of participants included in the present study was 50; 25 had T2DM (19 males and 6 females) and 25 were non-diabetic (18 males and 7 females). The number of STIs placed in T2DM patients was 41, Whereas in non-diabetic patients it was 38. The age of subjects having T2DM ranged from 60 ± 4.5 years. Whereas the mean age of non-diabetic participants was 61 ± 3.8. Among T2DM patients, 79.9% had a family genetic history whereas 40.1% didn’t show any family history of the disease. Moreover, patients with T2DM had levels of HbA1c mean ± SD 8.7 ± 1.4. Participants who were not diabetic had HbA1c levels of 4.6 ± 0.3.

In both groups, the major reason for loss of teeth was caries followed by a periodontal condition. Posterior to the maxillary second molar a total of 79 STIs were placed in both groups. Implant duration in T2DM participants was 82.4 ± 10.5 months and in non-diabetics it was 76.8 ± 13.9 months. Similarly, the mean loading period in T2DM in months was 3.8 ± 0.6, and in non-diabetics it was 4.1 ± 0.4. When inquiring about restoration type in both groups, the primary method used was screw-retaining. When inquiring about the status of oral hygiene and brushing techniques, most of the participants in each group brushed their teeth once daily. Moreover, improved dental visits were also found in non-diabetic patients (Table 1).

### 3.2. Periodontal and Radiographic Peri-Implant Parameters in a Cohort at 12 to 60 Months Follow-Up

Table 2 shows periodontal parameters around STIs in participants with diabetes and non-diabetes at 12 months and 60 months of follow-up. At 1 year follow-up, the mean PI % range in T2DM participants was 18.9% (19.2–21.4%) and in non-diabetics the proportion was 17.6% (16.3–18.5%). Mean PD in millimeters around STIs in T2DM patients at 12 months follow-up was recorded at 4.2 mm (1.3 ± 5.0) and in non-diabetics it was found at around 2.5 mm (1.1 ± 3.2). BoP in diabetics was 44.9% (39.8–46.4%) and in non-diabetics it was 22.8% (17.2–24.6%). Similarly, when the same parameters were observed at 60 months of follow-up, the mean PI% range in T2DM participants was 26.18% (25.4–29.1%) and non-diabetics it was 24.42% (20.1–25.5%). Similarly, mean PD in millimeters around STIs in T2DM patients at 5 years was observed to be 4.4 mm (2.3 ± 4.8) and in non-diabetic patients it was 2.9 mm (1.4 ± 3.4). In addition, BoP in diabetic participants was 39.54% (27.7–42.1%) and non-diabetics it was 24.42% (20.1–25.5%).

The radiographic parameters showed that CBL around STIs in T2DM patients was 1.4 ± 0.4 and 0.6 ± 0.2 mm in non-diabetics at 12 months of follow-up. Similarly, at 5 years of follow-up, CBL in T2DM patients was 2.8 ± 0.4 mm and 1.4 ± 0.3 mm in non-diabetics. Periodontal and radiographic parameters, i.e., BoP, PD, and CBL, showed a statistically significant increase in STIs in diabetics than in non-diabetics at 12 and 60 months (*p* < 0.05). However, PI showed no significant difference in both groups (*p* > 0.05) (Table 2) in an intra-group comparison at 12 months and 60 months follow-up, as shown in the results of T2DM and non- diabetic patients (*p* < 0.05) (Table 2).

### 3.3. Restorative Parameters

A total of six STIs failed, i.e., two in the non-diabetic and four in the T2DM group. The failure was reported due to the following reason: T2DM patients lack osseointegration and implant loosening (*n* = 2). Among T2DM patients, chipping of the ceramic and fracture of the framework were also noted (*n* = 2). In non-diabetics, the loosening of the abutment screw failed (*n* = 2).

## 4. Discussion

The present study was based on the postulation that STI will be a viable option in both T2DM and nondiabetic patients. However, the present study showed that diabetics have poor periodontal peri-implant parameters (BoP, PD) and radiographic parameters (CBL), in comparison to non-diabetics. Therefore, the proposed hypothesis was rejected. This hypothesis was based on previous work and clinical trials which reported a significant decline in periodontal parameters, periimplantitis, and loss of alveolar bone with paresthesia around dental implants among diabetics when compared to non-diabetics [22,28,29,30]. The outcomes of in-vivo studies reported that among participants with T2DM the intensity of parameters assessed is predisposed by glycemic status. Moreover, the success of the implant is dependent on moderate glycemic control [17,31]. Some studies have reported high levels of inflammatory cytokines (IL-β and TNF-α) in crevicular fluids of diabetic participants impairing new bone formation and weakening the healing process [22,32].

Chronic hyperglycemia is responsible for advanced glycation end products (AGEs) throughout the body specifically in the soft tissues of the oral cavity. With the interaction of AGEs with its receptors, there is an increase in inflammation and a hike in pro-inflammatory cytokines in body fluid (serum, follicular fluid, and saliva). In the present study, since the Hb1Ac levels were higher in diabetic patients it is estimated that it is more likely that there was a greater interaction of AGEs with AGE receptors, compromising periodontal (BOP, and PD) and restorative per-implant parameters (CBL). Abundant plaque is claimed to be a prime pathological process of peri-implant and periodontal disease, resulting in per-implant inflammation and ensuing in the formation of deep PD and rising BoP. Other factors that contribute to an increase in PD are chronic continuous infection due to changes in immunology, impaired function of polymorphonuclears compromising the blood flow, and delaying healing [32,33].

BoP can be considered the sole indicator of both periodontal and per-implant inflammation. In the present study, peri-implant parameters (PD, BoP) showed a significant increase from 12 to 60 months of follow-up in diabetic participants compared to non-diabetics. Increased BoP in T2DM can be attributed to basement membrane thickening due to the process of glycosylation of membrane protein, which results in capillary thickening, decreasing the diffusion of oxygen to gingival tissue [34]. Moreover, a recent study by Witzum et al. [35] noted micro- and macrostructure alterations due to the excessive production of cytokine and growth factors.

In the present study, radiographic parameters were evaluated through CBL. Higher peri-implant CBL was observed among diabetics compared to nondiabetics around STIs. Increased resorption and CBL in T2DM can be credited to augmented proinflammatory cytokines such as interleukin *(IL)-6* and *IL-1β,* matrix metalloproteinase (*MMP*), and TNF-*α* in the peri-implant sulcular fluid (PISF) [36,37]. Increased levels of cytokines alter the bone physiology, supplementing osteoclastic activity and reducing osteoblastic action [38]. This reduces the bone turnover, hence promoting marginal bone loss. For this reason, it is important to balance, control and maintain the level of glycemia to promote the overall general care of patients with T2DM [38,39]. Other factors that influence bone health and growth are the maintenance of good oral hygiene, duration of disease, and age. Increasing age is considered to be a predisposing factor for bone loss. Al-Sowygh et al. reported minimum bone loss around implants in younger adults [14]. Since the study was performed on T2DM participants having an average age of >60 years, both factors may have contributed to the higher resorption of crestal bone in diabetics with STI. Confounding factors, i.e., C/I ratio and occlusal overload, were not assessed, which may also influence CBL [40,41].

It is well-established that for patients with chronic disease, regular dental and general health checkups along with sustaining good hygiene play a pivotal role in the maintenance and decrease of the progression of the disease [42]. Specifically for individuals with T2DM, adequate glycemic control along with regular dental visits are of utmost importance. However, in the existing study, patients with T2DM utilized dental care less compared to non-diabetics.

It is important to recognize the limitations of the present study. Males were almost twice as represented in number as compared to females in the present study. As hormonal changes and postmenopausal status influence bone metabolism in females, a balanced distribution of gender among study participants would show different peri-implant outcomes. Moreover, the peri-implant pro-inflammatory cytokine profile was not evaluated in the present study. Levels of cytokines around implants would provide a better insight and explanation of the inflammatory process around STIs.

Therefore, future studies should be focused on the investigation of peri-implant crevicular fluid (PICF) proinflammatory and bone metabolism cytokine profiles in STI patients with and without T2DM. Microbiological assessment of plaque should also be performed around STIs for a better understanding of microbial pathology to plan targeted therapeutic approaches for the successful clinical application of STIs.

## 5. Conclusions

Patients restored with STI having T2DM had poor peri-implant inflammatory (BoP, PD, CBL) and restorative parameters at 12 and 60 months compared to healthy (non-diabetic) patients. It appears that STIs show a poorer prognosis among diabetics than healthy individuals. For the long-term functional stability of STI, glycemic and plaque control are critical factors for clinical success.

## Figures and Tables

**Table 1:** General characteristics of the cohort (t-test).

*Characteristics*	*T2DM (n)*	*Non-Diabetic (n)*
Male/female Patients (*n*)	19/6 *n* = 25	18/7 *n* = 25
Age (years)	60 ± 4.5	61 ± 3.8
HbA1c (mean ± SD)%	8.7 ± 1.4	4.6 ± 0.3
Family history of diabetes %	79.9	40.1
**Reason for missed tooth %**		
*Caries*	81	73
*Periodontal disease*	19	27
*Trauma*	0	0
Total number of implants	41	38
Loading of an implant (month ± SD)	3.8 ± 0.6	4.1 ± 0.4
Implant duration (months)	82.4 ± 10.5	76.8 ± 13.9
**Type of restoration**		
*Screw retained*	30	34
*Cemented*	11	4
**Brushing**		
*Once daily*	22	18
*Twice daily*	3	6
Number of dental visits	3	6

T T2DM (type 2 diabetes mellitus).

**Table 2:** Periodontal parameters and radiographic peri-implant indices at 12 months and 60 months around STIs in patients with diabetes and non-diabetes using the Kruskal–Wallis test.

*Periodontal Parameters*	*12-Month Follow-Up* *N = 25 Each*		*60 Months Follow-Up* *N = 25 Each*		*p-Value*
	*T2DM* *Mean (Min–Max)*	*Non-Diabetic* *Mean (Min–Max)*	*T2DM* *Mean (Min–Max)*	*Nondiabetic* *Mean (Min–Max)*	
*Mean BoP % range*	44.9 (39.8–46.4)	22.8± (17.2–24.6)	39.54 (27.7–42.1)	27.4 (22.9–31.6)	0.031 *
*Mean PD mm* *in range*	4.2 (1.3 ± 5.0)	2.5 (1.1 ± 3.2)	4.4 (2.3 ± 4.8)	2.9 (1.4 ± 3.4)	0.020 *
*Mean PI % range*	18.9 (19.2–21.4)	17.6 (16.3–18.5)	26.18 (25.4–29.1)	24.42 (20.1–25.5)	**0.211**
*CBL in mm*	1.4 ± 0.4	0.6 ± 0.2	2.8 ± 0.4	1.4 ± 0.3	0.044 *

* Statistically significant difference between groups; bleeding on probing (BoP); probing depth (PD) plaque index (PI) and crestal bone loss (CBL). Intra-group comparison at 12 month and 60 month follow-up results of T2DM and non-diabetic patients (*p* < 0.05).

## Data Availability

The data is available on contact from the corresponding author.
